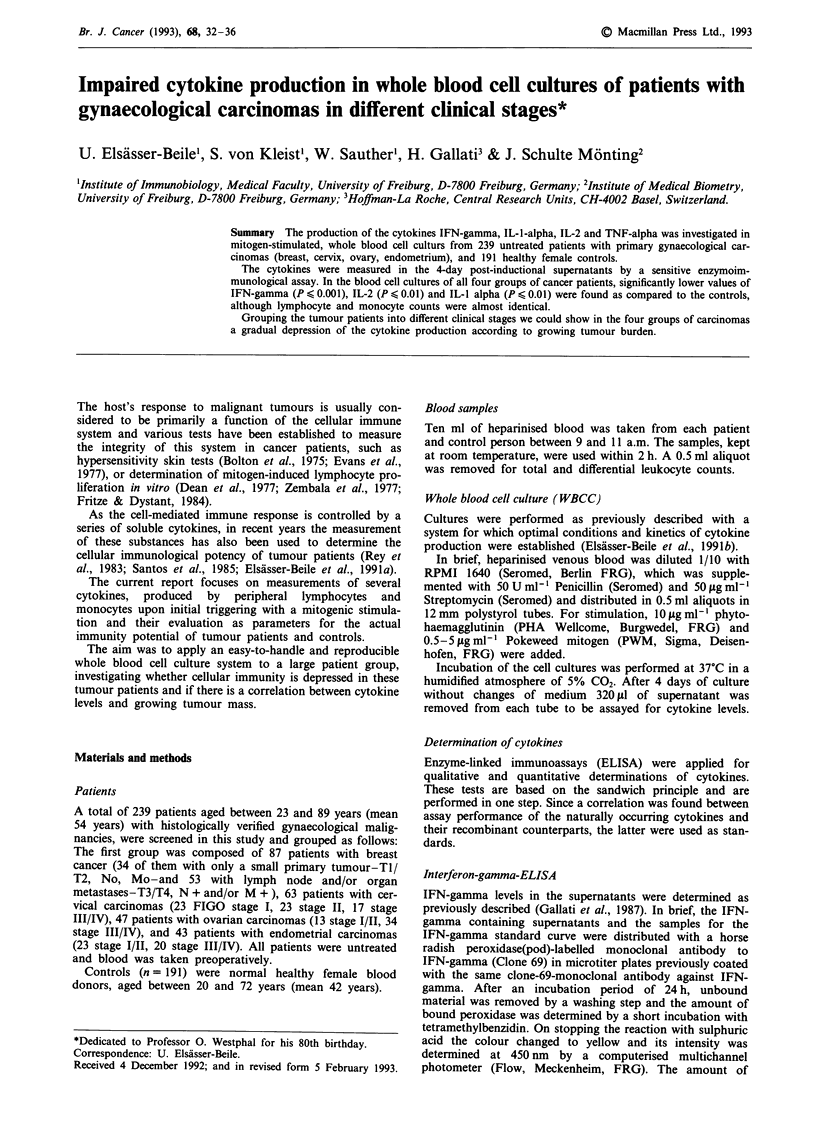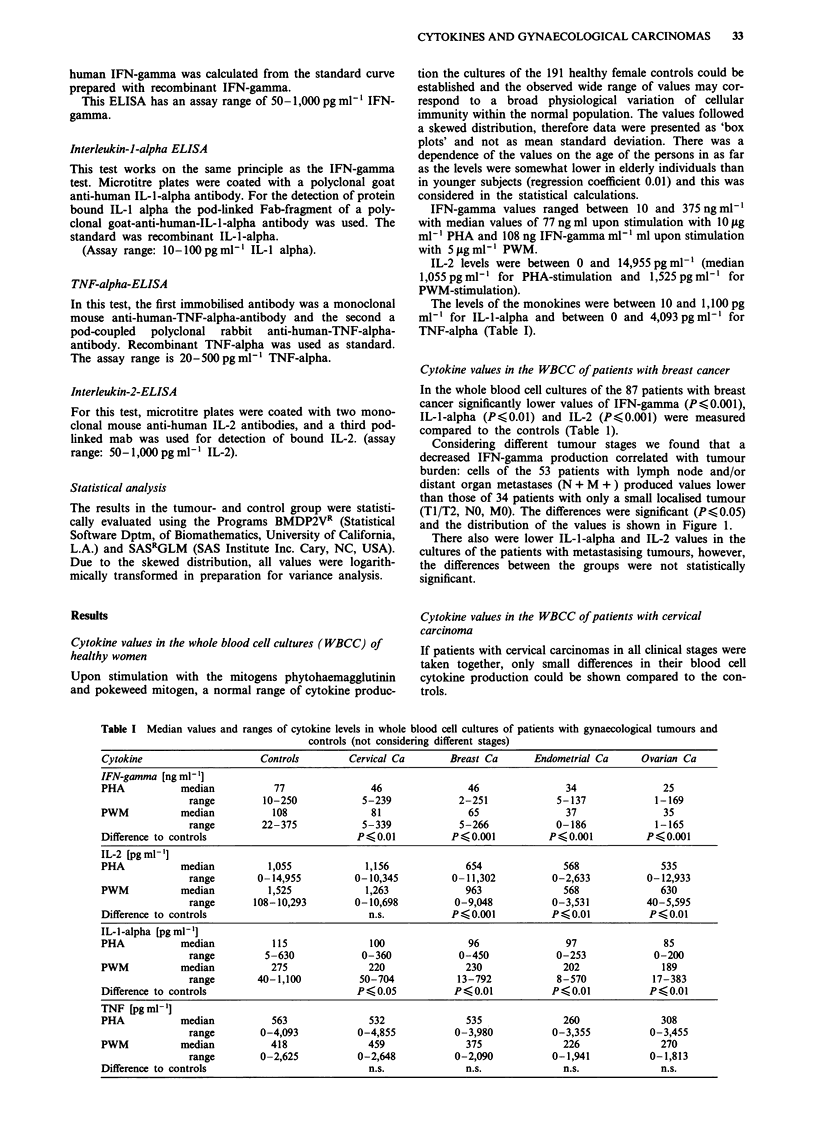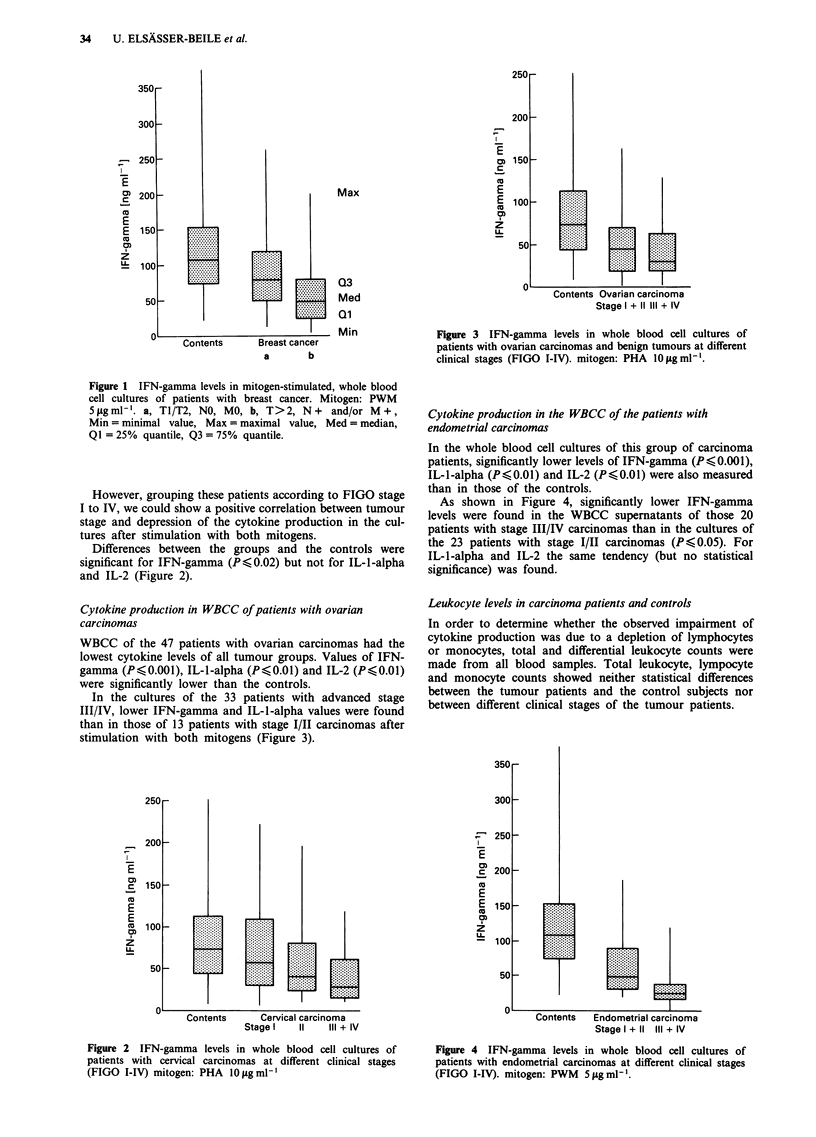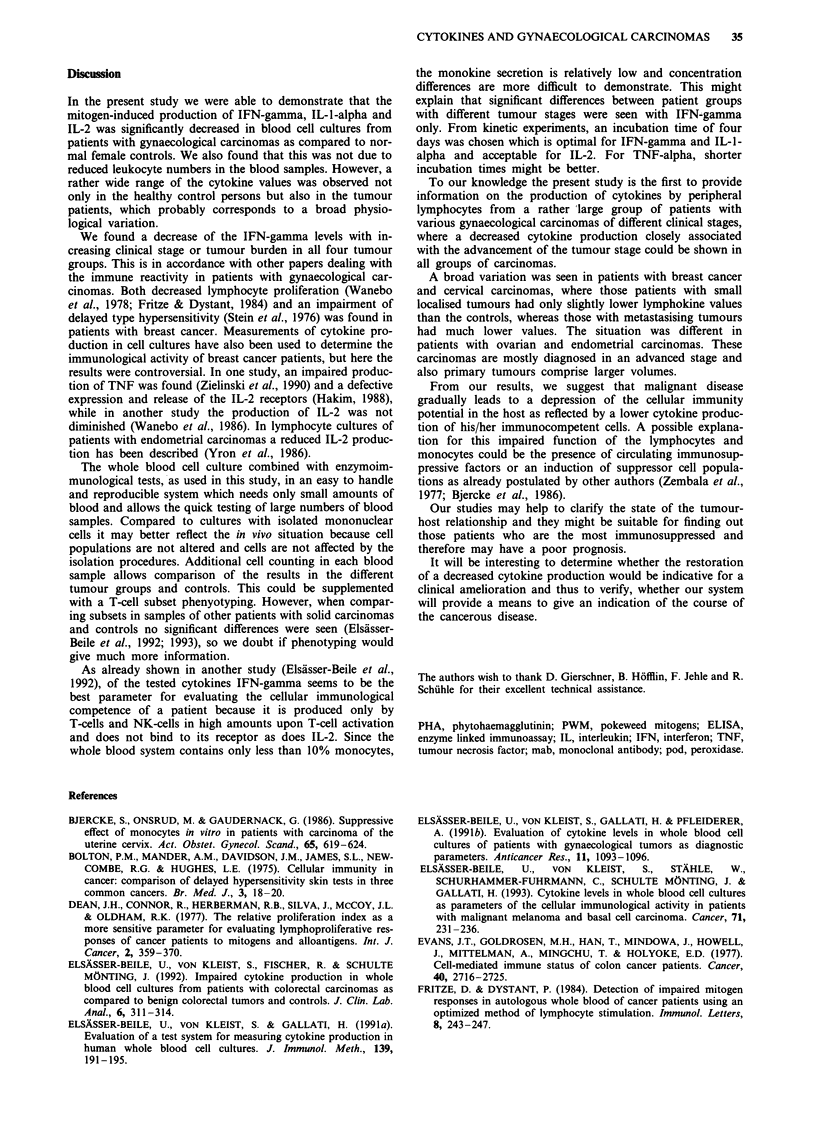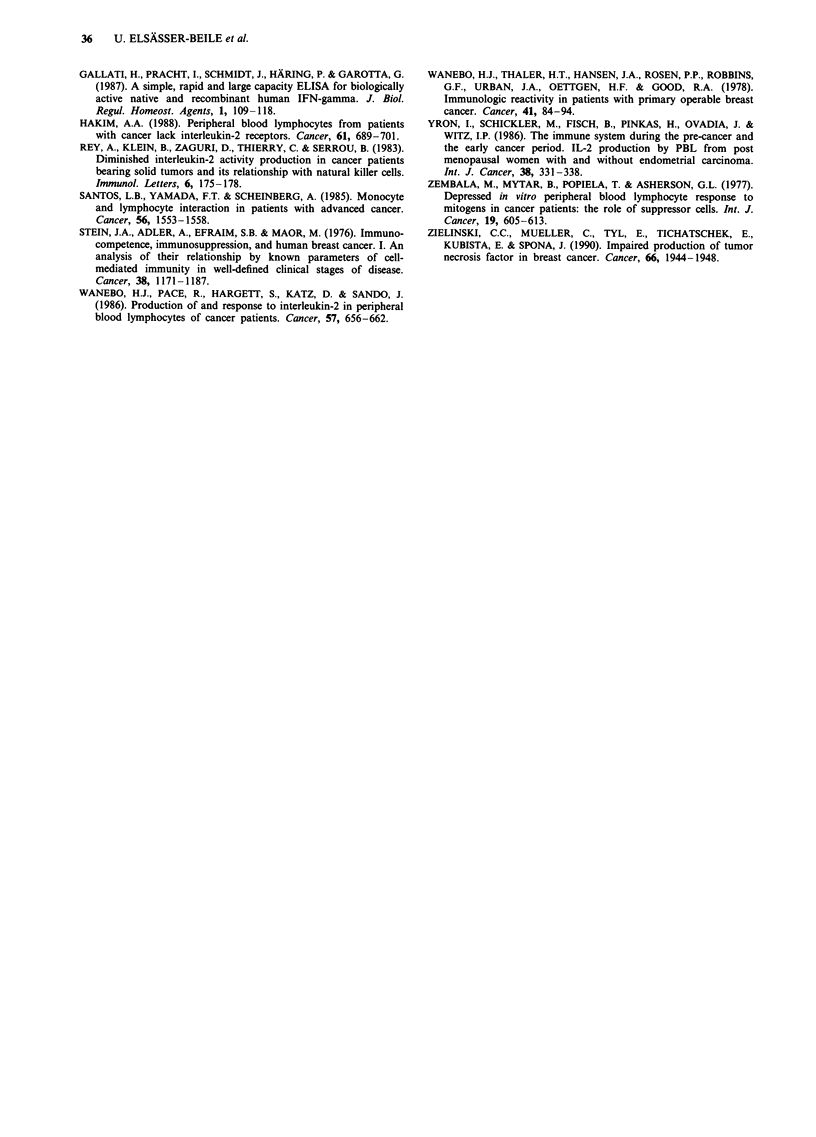# Impaired cytokine production in whole blood cell cultures of patients with gynaecological carcinomas in different clinical stages.

**DOI:** 10.1038/bjc.1993.282

**Published:** 1993-07

**Authors:** U. Elsässer-Beile, S. von Kleist, W. Sauther, H. Gallati, J. S. Mönting

**Affiliations:** Institute of Immunobiology, Medical Faculty, University of Freiburg, Germany.

## Abstract

The production of the cytokines IFN-gamma, IL-1-alpha, IL-2 and TNF-alpha was investigated in mitogen-stimulated, whole blood cell culture from 239 untreated patients with primary gynaecological carcinomas (breast, cervix, ovary, endometrium), and 191 healthy female controls. The cytokines were measured in the 4-day post-induction supernatants by a sensitive enzymoimmunological assay. In the blood cell cultures of all four groups of cancer patients, significantly lower values of IFN-gamma (P < or = 0.001), IL-2 (P < or = 0.01) and IL-1 alpha (P < or = 0.01) were found as compared to the controls, although lymphocyte and monocyte counts were almost identical. Grouping the tumour patients into different clinical stages we could show in the four groups of carcinomas a gradual depression of the cytokine production according to growing tumour burden.


 © Macmillan Press Ltd., 1993

### Impaired cytokine production in whole blood cell cultures of patients with gynaecological carcinomas in different clinical stages*

U. Elsässer-Beile[1], S. von Kleist[1], W. Sauther[1], H. Gallati[3] & J. Schulte Mönting[2]

[1]*Institute of Immunobiology, Medical Faculty, University of Freiburg, D-7800 Freiburg, Germany;* [2]*Institute of Medical Biometry, University of Freiburg, D-7800 Freiburg, Germany;* [3]*Hoffman-La Roche, Central Research Units, CH-4002 Basel, Switzerland.*

**Summary** The production of the cytokines IFN-gamma, IL-1-alpha, IL-2 and TNF-alpha was investigated in mitogen-stimulated, whole blood cell culturs from 239 untreated patients with primary gynaecological carcinomas (breast, cervix, ovary, endometrium), and 191 healthy female controls.

The cytokines were measured in the 4-day post-inductional supernatants by a sensitive enzymoimmunological assay. In the blood cell cultures of all four groups of cancer patients, significantly lower values of IFN-gamma ($P \leqslant 0.001$), IL-2 ($P \leqslant 0.01$) and IL-1 alpha ($P \leqslant 0.01$) were found as compared to the controls, although lymphocyte and monocyte counts were almost identical.

Grouping the tumour patients into different clinical stages we could show in the four groups of carcinomas a gradual depression of the cytokine production according to growing tumour burden.

The host's response to malignant tumours is usually considered to be primarily a function of the cellular immune system and various tests have been established to measure the integrity of this system in cancer patients, such as hypersensitivity skin tests (Bolton *et al.*, 1975; Evans *et al.*, 1977), or determination of mitogen-induced lymphocyte proliferation *in vitro* (Dean *et al.*, 1977; Zembala *et al.*, 1977; Fritze & Dystant, 1984).

As the cell-mediated immune response is controlled by a series of soluble cytokines, in recent years the measurement of these substances has also been used to determine the cellular immunological potency of tumour patients (Rey *et al.*, 1983; Santos *et al.*, 1985; Elsässer-Beile *et al.*, 1991*a*).

The current report focuses on measurements of several cytokines, produced by peripheral lymphocytes and monocytes upon initial triggering with a mitogenic stimulation and their evaluation as parameters for the actual immunity potential of tumour patients and controls.

The aim was to apply an easy-to-handle and reproducible whole blood cell culture system to a large patient group, investigating whether cellular immunity is depressed in these tumour patients and if there is a correlation between cytokine levels and growing tumour mass.

### Materials and methods

### *Patients*

A total of 239 patients aged between 23 and 89 years (mean 54 years) with histologically verified gynaecological malignancies, were screened in this study and grouped as follows: The first group was composed of 87 patients with breast cancer (34 of them with only a small primary tumour-T1/T2, No, Mo-and 53 with lymph node and/or organ metastases-T3/T4, N + and/or M + ), 63 patients with cervical carcinomas (23 FIGO stage I, 23 stage II, 17 stage III/IV), 47 patients with ovarian carcinomas (13 stage I/II, 34 stage III/IV), and 43 patients with endometrial carcinomas (23 stage I/II, 20 stage III/IV). All patients were untreated and blood was taken preoperatively.

Controls ($n = 191$) were normal healthy female blood donors, aged between 20 and 72 years (mean 42 years).

### *Blood samples*

Ten ml of heparinised blood was taken from each patient and control person between 9 and 11 a.m. The samples, kept at room temperature, were used within 2 h. A 0.5 ml aliquot was removed for total and differential leukocyte counts.

### *Whole blood cell culture (WBCC)*

Cultures were performed as previously described with a system for which optimal conditions and kinetics of cytokine production were established (Elsässer-Beile *et al.*, 1991*b*).

In brief, heparinised venous blood was diluted 1/10 with RPMI 1640 (Seromed, Berlin FRG), which was supplemented with 50 U $ml^{-1}$ Penicillin (Seromed) and 50 $\mu g\, ml^{-1}$ Streptomycin (Seromed) and distributed in 0.5 ml aliquots in 12 mm polystyrol tubes. For stimulation, 10 $\mu g\, ml^{-1}$ phytohaemagglutinin (PHA Wellcome, Burgwedel, FRG) and 0.5-5 $\mu g\, ml^{-1}$ Pokeweed mitogen (PWM, Sigma, Deisenhofen, FRG) were added.

Incubation of the cell cultures was performed at 37°C in a humidified atmosphere of 5% $CO_2$. After 4 days of culture without changes of medium 320 µl of supernatant was removed from each tube to be assayed for cytokine levels.

### *Determination of cytokines*

Enzyme-linked immunoassays (ELISA) were applied for qualitative and quantitative determinations of cytokines. These tests are based on the sandwich principle and are performed in one step. Since a correlation was found between assay performance of the naturally occurring cytokines and their recombinant counterparts, the latter were used as standards.

### *Interferon-gamma-ELISA*

IFN-gamma levels in the supernatants were determined as previously described (Gallati *et al.*, 1987). In brief, the IFN-gamma containing supernatants and the samples for the IFN-gamma standard curve were distributed with a horse radish peroxidase(pod)-labelled monoclonal antibody to IFN-gamma (Clone 69) in microtiter plates previously coated with the same clone-69-monoclonal antibody against IFN-gamma. After an incubation period of 24 h, unbound material was removed by a washing step and the amount of bound peroxidase was determined by a short incubation with tetramethylbenzidin. On stopping the reaction with sulphuric acid the colour changed to yellow and its intensity was determined at 450 nm by a computerised multichannel photometer (Flow, Meckenheim, FRG). The amount of

*Dedicated to Professor O. Westphal for his 80th birthday.
Correspondence: U. Elsässer-Beile.
Received 4 December 1992; and in revised form 5 February 1993.

human IFN-gamma was calculated from the standard curve prepared with recombinant IFN-gamma.

This ELISA has an assay range of 50–1,000 pg $ml^{-1}$ IFN-gamma.

### *Interleukin-1-alpha ELISA*

This test works on the same principle as the IFN-gamma test. Microtitre plates were coated with a polyclonal goat anti-human IL-1-alpha antibody. For the detection of protein bound IL-1 alpha the pod-linked Fab-fragment of a polyclonal goat-anti-human-IL-1-alpha antibody was used. The standard was recombinant IL-1-alpha.

(Assay range: 10–100 pg $ml^{-1}$ IL-1 alpha).

### *TNF-alpha-ELISA*

In this test, the first immobilised antibody was a monoclonal mouse anti-human-TNF-alpha-antibody and the second a pod-coupled polyclonal rabbit anti-human-TNF-alpha-antibody. Recombinant TNF-alpha was used as standard. The assay range is 20–500 pg $ml^{-1}$ TNF-alpha.

### *Interleukin-2-ELISA*

For this test, microtitre plates were coated with two monoclonal mouse anti-human IL-2 antibodies, and a third pod-linked mab was used for detection of bound IL-2. (assay range: 50–1,000 pg $ml^{-1}$ IL-2).

### *Statistical analysis*

The results in the tumour- and control group were statistically evaluated using the Programs BMDP2V$^R$ (Statistical Software Dptm, of Biomathematics, University of California, L.A.) and SAS$^R$GLM (SAS Institute Inc. Cary, NC, USA). Due to the skewed distribution, all values were logarithmically transformed in preparation for variance analysis.

### Results

### *Cytokine values in the whole blood cell cultures (WBCC) of healthy women*

Upon stimulation with the mitogens phytohaemagglutinin and pokeweed mitogen, a normal range of cytokine production the cultures of the 191 healthy female controls could be established and the observed wide range of values may correspond to a broad physiological variation of cellular immunity within the normal population. The values followed a skewed distribution, therefore data were presented as 'box plots' and not as mean standard deviation. There was a dependence of the values on the age of the persons in as far as the levels were somewhat lower in elderly individuals than in younger subjects (regression coefficient 0.01) and this was considered in the statistical calculations.

IFN-gamma values ranged between 10 and 375 ng $ml^{-1}$ with median values of 77 ng ml upon stimulation with 10 μg $ml^{-1}$ PHA and 108 ng IFN-gamma $ml^{-1}$ ml upon stimulation with 5 μg $ml^{-1}$ PWM.

IL-2 levels were between 0 and 14,955 pg $ml^{-1}$ (median 1,055 pg $ml^{-1}$ for PHA-stimulation and 1,525 pg $ml^{-1}$ for PWM-stimulation).

The levels of the monokines were between 10 and 1,100 pg $ml^{-1}$ for IL-1-alpha and between 0 and 4,093 pg $ml^{-1}$ for TNF-alpha (Table I).

### *Cytokine values in the WBCC of patients with breast cancer*

In the whole blood cell cultures of the 87 patients with breast cancer significantly lower values of IFN-gamma ($P \leqslant 0.001$), IL-1-alpha ($P \leqslant 0.01$) and IL-2 ($P \leqslant 0.001$) were measured compared to the controls (Table 1).

Considering different tumour stages we found that a decreased IFN-gamma production correlated with tumour burden: cells of the 53 patients with lymph node and/or distant organ metastases (N + M + ) produced values lower than those of 34 patients with only a small localised tumour (T1/T2, N0, M0). The differences were significant ($P \leqslant 0.05$) and the distribution of the values is shown in Figure 1.

There also were lower IL-1-alpha and IL-2 values in the cultures of the patients with metastasising tumours, however, the differences between the groups were not statistically significant.

### *Cytokine values in the WBCC of patients with cervical carcinoma*

If patients with cervical carcinomas in all clinical stages were taken together, only small differences in their blood cell cytokine production could be shown compared to the controls.

**Table I** Median values and ranges of cytokine levels in whole blood cell cultures of patients with gynaecological tumours and controls (not considering different stages)

| *Cytokine* | | *Controls* | *Cervical Ca* | *Breast Ca* | *Endometrial Ca* | *Ovarian Ca* |
|---|---|---|---|---|---|---|
| *IFN-gamma* [ng $ml^{-1}$] | | | | | | |
| PHA | median | 77 | 46 | 46 | 34 | 25 |
| | range | 10–250 | 5–239 | 2–251 | 5–137 | 1–169 |
| PWM | median | 108 | 81 | 65 | 37 | 35 |
| | range | 22–375 | 5–339 | 5–266 | 0–186 | 1–165 |
| Difference to controls | | | $P \leqslant 0.01$ | $P \leqslant 0.001$ | $P \leqslant 0.001$ | $P \leqslant 0.001$ |
| IL-2 [pg $ml^{-1}$] | | | | | | |
| PHA | median | 1,055 | 1,156 | 654 | 568 | 535 |
| | range | 0–14,955 | 0–10,345 | 0–11,302 | 0–2,633 | 0–12,933 |
| PWM | median | 1,525 | 1,263 | 963 | 568 | 630 |
| | range | 108–10,293 | 0–10,698 | 0–9,048 | 0–3,531 | 40–5,595 |
| Difference to controls | | | n.s. | $P \leqslant 0.001$ | $P \leqslant 0.01$ | $P \leqslant 0.01$ |
| IL-1-alpha [pg $ml^{-1}$] | | | | | | |
| PHA | median | 115 | 100 | 96 | 97 | 85 |
| | range | 5–630 | 0–360 | 0–450 | 0–253 | 0–200 |
| PWM | median | 275 | 220 | 230 | 202 | 189 |
| | range | 40–1,100 | 50–704 | 13–792 | 8–570 | 17–383 |
| Difference to controls | | | $P \leqslant 0.05$ | $P \leqslant 0.01$ | $P \leqslant 0.01$ | $P \leqslant 0.01$ |
| TNF [pg $ml^{-1}$] | | | | | | |
| PHA | median | 563 | 532 | 535 | 260 | 308 |
| | range | 0–4,093 | 0–4,855 | 0–3,980 | 0–3,355 | 0–3,455 |
| PWM | median | 418 | 459 | 375 | 226 | 270 |
| | range | 0–2,625 | 0–2,648 | 0–2,090 | 0–1,941 | 0–1,813 |
| Difference to controls | | | n.s. | n.s. | n.s. | n.s. |

**Figure 1** IFN-gamma levels in mitogen-stimulated, whole blood cell cultures of patients with breast cancer. Mitogen: PWM 5 µg ml$^{-1}$. **a**, T1/T2, N0, M0, **b**, T>2, N+ and/or M+, Min = minimal value, Max = maximal value, Med = median, Q1 = 25% quantile, Q3 = 75% quantile.

However, grouping these patients according to FIGO stage I to IV, we could show a positive correlation between tumour stage and depression of the cytokine production in the cultures after stimulation with both mitogens.

Differences between the groups and the controls were significant for IFN-gamma ($P \leqslant 0.02$) but not for IL-1-alpha and IL-2 (Figure 2).

*Cytokine production in WBCC of patients with ovarian carcinomas*

WBCC of the 47 patients with ovarian carcinomas had the lowest cytokine levels of all tumour groups. Values of IFN-gamma ($P \leqslant 0.001$), IL-1-alpha ($P \leqslant 0.01$) and IL-2 ($P \leqslant 0.01$) were significantly lower than the controls.

In the cultures of the 33 patients with advanced stage III/IV, lower IFN-gamma and IL-1-alpha values were found than in those of 13 patients with stage I/II carcinomas after stimulation with both mitogens (Figure 3).

**Figure 3** IFN-gamma levels in whole blood cell cultures of patients with ovarian carcinomas and benign tumours at different clinical stages (FIGO I-IV). mitogen: PHA 10 µg ml$^{-1}$.

*Cytokine production in the WBCC of the patients with endometrial carcinomas*

In the whole blood cell cultures of this group of carcinoma patients, significantly lower levels of IFN-gamma ($P \leqslant 0.001$), IL-1-alpha ($P \leqslant 0.01$) and IL-2 ($P \leqslant 0.01$) were also measured than in those of the controls.

As shown in Figure 4, significantly lower IFN-gamma levels were found in the WBCC supernatants of those 20 patients with stage III/IV carcinomas than in the cultures of the 23 patients with stage I/II carcinomas ($P \leqslant 0.05$). For IL-1-alpha and IL-2 the same tendency (but no statistical significance) was found.

*Leukocyte levels in carcinoma patients and controls*

In order to determine whether the observed impairment of cytokine production was due to a depletion of lymphocytes or monocytes, total and differential leukocyte counts were made from all blood samples. Total leukocyte, lympocyte and monocyte counts showed neither statistical differences between the tumour patients and the control subjects nor between different clinical stages of the tumour patients.

**Figure 2** IFN-gamma levels in whole blood cell cultures of patients with cervical carcinomas at different clinical stages (FIGO I-IV) mitogen: PHA 10 µg ml$^{-1}$

**Figure 4** IFN-gamma levels in whole blood cell cultures of patients with endometrial carcinomas at different clinical stages (FIGO I-IV). mitogen: PWM 5 µg ml$^{-1}$.

### Discussion

In the present study we were able to demonstrate that the mitogen-induced production of IFN-gamma, IL-1-alpha and IL-2 was significantly decreased in blood cell cultures from patients with gynaecological carcinomas as compared to normal female controls. We also found that this was not due to reduced leukocyte numbers in the blood samples. However, a rather wide range of the cytokine values was observed not only in the healthy control persons but also in the tumour patients, which probably corresponds to a broad physiological variation.

We found a decrease of the IFN-gamma levels with increasing clinical stage or tumour burden in all four tumour groups. This is in accordance with other papers dealing with the immune reactivity in patients with gynaecological carcinomas. Both decreased lymphocyte proliferation (Wanebo *et al.*, 1978; Fritze & Dystant, 1984) and an impairment of delayed type hypersensitivity (Stein *et al.*, 1976) was found in patients with breast cancer. Measurements of cytokine production in cell cultures have also been used to determine the immunological activity of breast cancer patients, but here the results were controversial. In one study, an impaired production of TNF was found (Zielinski *et al.*, 1990) and a defective expression and release of the IL-2 receptors (Hakim, 1988), while in another study the production of IL-2 was not diminished (Wanebo *et al.*, 1986). In lymphocyte cultures of patients with endometrial carcinomas a reduced IL-2 production has been described (Yron *et al.*, 1986).

The whole blood cell culture combined with enzymoimmunological tests, as used in this study, in an easy to handle and reproducible system which needs only small amounts of blood and allows the quick testing of large numbers of blood samples. Compared to cultures with isolated mononuclear cells it may better reflect the *in vivo* situation because cell populations are not altered and cells are not affected by the isolation procedures. Additional cell counting in each blood sample allows comparison of the results in the different tumour groups and controls. This could be supplemented with a T-cell subset phenyotyping. However, when comparing subsets in samples of other patients with solid carcinomas and controls no significant differences were seen (Elsässer-Beile *et al.*, 1992; 1993), so we doubt if phenotyping would give much more information.

As already shown in another study (Elsässer-Beile *et al.*, 1992), of the tested cytokines IFN-gamma seems to be the best parameter for evaluating the cellular immunological competence of a patient because it is produced only by T-cells and NK-cells in high amounts upon T-cell activation and does not bind to its receptor as does IL-2. Since the whole blood system contains only less than 10% monocytes, the monokine secretion is relatively low and concentration differences are more difficult to demonstrate. This might explain that significant differences between patient groups with different tumour stages were seen with IFN-gamma only. From kinetic experiments, an incubation time of four days was chosen which is optimal for IFN-gamma and IL-1-alpha and acceptable for IL-2. For TNF-alpha, shorter incubation times might be better.

To our knowledge the present study is the first to provide information on the production of cytokines by peripheral lymphocytes from a rather large group of patients with various gynaecological carcinomas of different clinical stages, where a decreased cytokine production closely associated with the advancement of the tumour stage could be shown in all groups of carcinomas.

A broad variation was seen in patients with breast cancer and cervical carcinomas, where those patients with small localised tumours had only slightly lower lymphokine values than the controls, whereas those with metastasising tumours had much lower values. The situation was different in patients with ovarian and endometrial carcinomas. These carcinomas are mostly diagnosed in an advanced stage and also primary tumours comprise larger volumes.

From our results, we suggest that malignant disease gradually leads to a depression of the cellular immunity potential in the host as reflected by a lower cytokine production of his/her immunocompetent cells. A possible explanation for this impaired function of the lymphocytes and monocytes could be the presence of circulating immunosuppressive factors or an induction of suppressor cell populations as already postulated by other authors (Zembala *et al.*, 1977; Bjercke *et al.*, 1986).

Our studies may help to clarify the state of the tumour-host relationship and they might be suitable for finding out those patients who are the most immunosuppressed and therefore may have a poor prognosis.

It will be interesting to determine whether the restoration of a decreased cytokine production would be indicative for a clinical amelioration and thus to verify, whether our system will provide a means to give an indication of the course of the cancerous disease.

The authors wish to thank D. Gierschner, B. Höfflin, F. Jehle and R. Schühle for their excellent technical assistance.

PHA, phytohaemagglutinin; PWM, pokeweed mitogens; ELISA, enzyme linked immunoassay; IL, interleukin; IFN, interferon; TNF, tumour necrosis factor; mab, monoclonal antibody; pod, peroxidase.

### References

BJERCKE, S., ONSRUD, M. & GAUDERNACK, G. (1986). Suppressive effect of monocytes *in vitro* in patients with carcinoma of the uterine cervix. *Act. Obstet. Gynecol. Scand.*, **65**, 619–624.

BOLTON, P.M., MANDER, A.M., DAVIDSON, J.M., JAMES, S.L., NEWCOMBE, R.G. & HUGHES, L.E. (1975). Cellular immunity in cancer: comparison of delayed hypersensitivity skin tests in three common cancers. *Br. Med. J.*, **3**, 18–20.

DEAN, J.H., CONNOR, R., HERBERMAN, R.B., SILVA, J., MCCOY, J.L. & OLDHAM, R.K. (1977). The relative proliferation index as a more sensitive parameter for evaluating lymphoproliferative responses of cancer patients to mitogens and alloantigens. *Int. J. Cancer*, **2**, 359–370.

ELSÄSSER-BEILE, U., VON KLEIST, S., FISCHER, R. & SCHULTE MÖNTING, J. (1992). Impaired cytokine production in whole blood cell cultures from patients with colorectal carcinomas as compared to benign colorectal tumors and controls. *J. Clin. Lab. Anal.*, **6**, 311–314.

ELSÄSSER-BEILE, U., VON KLEIST, S. & GALLATI, H. (1991*a*). Evaluation of a test system for measuring cytokine production in human whole blood cell cultures. *J. Immunol. Meth.*, **139**, 191–195.

ELSÄSSER-BEILE, U., VON KLEIST, S., GALLATI, H. & PFLEIDERER, A. (1991*b*). Evaluation of cytokine levels in whole blood cell cultures of patients with gynaecological tumors as diagnostic parameters. *Anticancer Res.*, **11**, 1093–1096.

ELSÄSSER-BEILE, U., VON KLEIST, S., STÄHLE, W., SCHURHAMMER-FUHRMANN, C., SCHULTE MÖNTING, J. & GALLATI, H. (1993). Cytokine levels in whole blood cell cultures as parameters of the cellular immunological activity in patients with malignant melanoma and basal cell carcinoma. *Cancer*, **71**, 231–236.

EVANS, J.T., GOLDROSEN, M.H., HAN, T., MINDOWA, J., HOWELL, J., MITTELMAN, A., MINGCHU, T. & HOLYOKE, E.D. (1977). Cell-mediated immune status of colon cancer patients. *Cancer*, **40**, 2716–2725.

FRITZE, D. & DYSTANT, P. (1984). Detection of impaired mitogen responses in autologous whole blood of cancer patients using an optimized method of lymphocyte stimulation. *Immunol. Letters*, **8**, 243–247.

GALLATI, H., PRACHT, I., SCHMIDT, J., HÄRING, P. & GAROTTA, G. (1987). A simple, rapid and large capacity ELISA for biologically active native and recombinant human IFN-gamma. *J. Biol. Regul. Homeost. Agents*, **1**, 109–118.

HAKIM, A.A. (1988). Peripheral blood lymphocytes from patients with cancer lack interleukin-2 receptors. *Cancer*, **61**, 689–701.

REY, A., KLEIN, B., ZAGURI, D., THIERRY, C. & SERROU, B. (1983). Diminished interleukin-2 activity production in cancer patients bearing solid tumors and its relationship with natural killer cells. *Immunol. Letters*, **6**, 175–178.

SANTOS, L.B., YAMADA, F.T. & SCHEINBERG, A. (1985). Monocyte and lymphocyte interaction in patients with advanced cancer. *Cancer*, **56**, 1553–1558.

STEIN, J.A., ADLER, A., EFRAIM, S.B. & MAOR, M. (1976). Immunocompetence, immunosuppression, and human breast cancer. I. An analysis of their relationship by known parameters of cell-mediated immunity in well-defined clinical stages of disease. *Cancer*, **38**, 1171–1187.

WANEBO, H.J., PACE, R., HARGETT, S., KATZ, D. & SANDO, J. (1986). Production of and response to interleukin-2 in peripheral blood lymphocytes of cancer patients. *Cancer*, **57**, 656–662.

WANEBO, H.J., THALER, H.T., HANSEN, J.A., ROSEN, P.P., ROBBINS, G.F., URBAN, J.A., OETTGEN, H.F. & GOOD, R.A. (1978). Immunologic reactivity in patients with primary operable breast cancer. *Cancer*, **41**, 84–94.

YRON, I., SCHICKLER, M., FISCH, B., PINKAS, H., OVADIA, J. & WITZ, I.P. (1986). The immune system during the pre-cancer and the early cancer period. IL-2 production by PBL from post menopausal women with and without endometrial carcinoma. *Int. J. Cancer*, **38**, 331–338.

ZEMBALA, M., MYTAR, B., POPIELA, T. & ASHERSON, G.L. (1977). Depressed *in vitro* peripheral blood lymphocyte response to mitogens in cancer patients: the role of suppressor cells. *Int. J. Cancer*, **19**, 605–613.

ZIELINSKI, C.C., MUELLER, C., TYL, E., TICHATSCHEK, E., KUBISTA, E. & SPONA, J. (1990). Impaired production of tumor necrosis factor in breast cancer. *Cancer*, **66**, 1944–1948.